# On the move

**DOI:** 10.7554/eLife.01414

**Published:** 2013-09-24

**Authors:** Robert A Forties, Jie Ma, Michelle D Wang

**Affiliations:** 1 **Robert A Forties** is in the Department of Physics-Laboratory of Atomic and Solid State Physics and the Howard Hughes Medical Institute, Cornell University, Ithaca, United Statesraf37@cornell.edu; 2 **Jie Ma** is in the Department of Physics-Laboratory of Atomic and Solid State Physics and the Howard Hughes Medical Institute, Cornell University, Ithaca, United Statesjm893@cornell.edu; 3 **Michelle D Wang** is in the Department of Physics-Laboratory of Atomic and Solid State Physics and the Howard Hughes Medical Institute, Cornell University, Ithaca, United Statesmdw17@cornell.edu

**Keywords:** RNA polymerase II, transcription elongation, Brownian ratchet, translocation, backtracking, Optical tweezers, *S. cerevisiae*

## Abstract

Single-molecule experiments have shed new light on the mechanisms responsible for the movement of RNA polymerase along DNA during transcription.

**Related research article** Dangkulwanich M, Ishibashi T, Liu S, Kireeva ML, Lubkowska L, Kashlev M, Bustamante CJ. 2013. Complete dissection of transcription elongation reveals slow translocation of RNA polymerase II in a linear ratchet mechanism. *eLife* **2**:e00971. doi: 10.7554/eLife.00971

**Image** Schematic of the dual optical trap used to measure the translocation of RNA polymerase

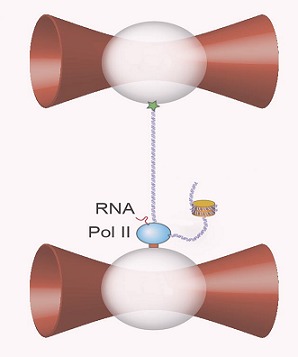

Transcription is a critical step in the series of events that results in genes being expressed as proteins. During transcription, an enzyme called RNA polymerase moves along a DNA template in order to produce a single strand of messenger RNA with a sequence of bases that matches the sequence in the DNA (with uracil replacing thymine). For several decades, a variety of structural, biochemical and single-molecule studies have been used to probe the details of transcription, but many open questions remain. Specifically, what are the key steps in the process that converts chemical energy into the mechanical energy that is needed to move the RNA polymerase in the required direction, and how fast are these steps? Now, in *eLife*, Carlos Bustamante and colleagues describe how a combination of single-molecule measurements and theoretical modelling has provided important insights into these questions (Dangkulwanich et al., 2013).

The main reaction pathway for transcription has three basic steps (Figure 1). First, the RNA polymerase moves forward by one base pair along the DNA template (a process called translocation); thermal energy actually causes the polymerase to oscillate back and forth, so something needs to happen to convert this random motion into motion along the DNA template in the correct direction. Second, a nucleotide triphosphate (NTP) molecule binds to the active site of the RNA polymerase. Third, chemical catalysis results in the nucleotide being added to the 3′ end of the RNA chain and pyrophosphate (which is produced when the phosphates are broken down to provide energy) being released. This whole process is then repeated to add another nucleotide. However, instead of moving forward overall, the RNA polymerase sometimes moves backwards along the DNA template and displaces the 3′ end of the RNA chain from the active site of the polymerase: this process, which is called backtracking, pauses RNA synthesis (Figure 1).

**Figure 1.**
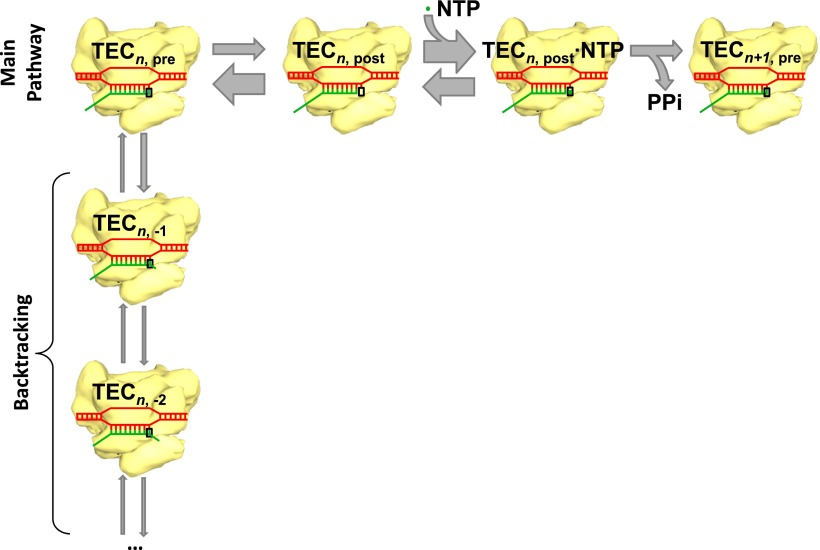
Transcription and backtracking. Active transcription (top left to right) involves an enzyme called RNA polymerase (yellow) moving forward along a DNA template (red) and adding nucleotides to one end of an RNA transcript (green). Alternatively, the RNA polymerase may enter the backtracking pathway (top left to bottom) which pauses the synthesis of RNA. The RNA polymerase is part of a larger structure called the transcription elongation complex (TEC) that comprises RNA polymerase, DNA and the nascent RNA transcript. The main reaction pathway for the addition of nucleotides has three steps, and it starts (top left) with the TEC in a ‘pre-translocated state’ and with *n* nucleotides in the RNA transcript. The first step involves the RNA polymerase moving to ‘post-translocated state’ (second from left). The second step involves a molecule of nucleotide triphosphate (NTP) binding to the active site of the RNA polymerase. The third step is a chemical reaction that results in the nucleotide (green dot) being added to the RNA transcript (to give *n*+1 nucleotides) and the TEC returning to a pre-translocated state; the energy needed to drive this reaction come from the nucleotide condensation reaction, with pyrophosphate (PPi) being released as a by-product of this process. The widths of the grey arrows are proportional to the rates of the various transitions reported by in Dangkulwanich et al.; it can be seen that the translocation steps are not in equilibrium, whereas the NTP binding steps are in equilibrium.

When developing theoretical models of the reaction pathway, researchers have generally assumed that the first two steps in the main reaction pathway—the translocation step and the NTP binding step—are much faster than the chemical catalysis step and may, therefore, be treated as though they are in equilibrium (Guajardo and Sousa, 1997; von Hippel and Pasman, 2002; Bai et al., 2004; Larson et al., 2012). However, this simple equilibrium model did not seem able to explain the results of experiments in which a force was applied to the RNA polymerase and the elongation velocity (the velocity at which the RNA chain is synthesized) was measured. This discrepancy prompted Steven Block of Stanford University and colleagues to propose the existence of a secondary site for binding NTP (Abbondanzieri et al., 2005; Larson et al., 2012).

There are good reasons to believe that NTP binding is in equilibrium: in other words, the rates at which NTP molecules bind to, and unbind from, the RNA polymerase are much faster than the catalysis rate. However, it is not clear that the rates at which the RNA polymerase moves forward and backward are also much faster than the catalysis rate. Now, by questioning the assumption that the translocation step is in equilibrium, Bustamante and colleagues—including Manchuta Dangkulwanich, Toyotaka Ishibashi and Shixin Liu as joint first authors—argue that a secondary NTP binding site is not needed.

Dangkulwanich et al. employed a dual-optical trapping technique (Galburt et al., 2007): one optical trap held a polystyrene bead that was attached to a molecule of RNA polymerase II (Pol II) from yeast, and the other trap held a bead that was attached to one end of the DNA template. Using this method, they were able to measure how the elongation velocity depended on the applied force and on the concentration of NTP, and also how pausing depended on these two variables. They also performed experiments in which a structure called a nucleosome was placed on the DNA template: this structure slows the forward motion of the Pol II, but it does not have any impact on the backward motion (Hodges et al., 2009).

Based on these results, Dangkulwanich et al. generated an energy landscape for the reaction pathway and arrived at several important conclusions. First, they found that the forward motion of the RNA polymerase was much slower than previously assumed, and also much slower than the backward motion: the forward motion was faster than the catalysis step by a factor of only 2.5, whereas the backwards motion was faster by a factor of 20. Since such a simple model with the translocation steps in the main reaction pathway not being in equilibrium could explain the force-velocity results, Dangkulwanich et al. argue that there is no need to invoke a more complex model with a secondary NTP binding site. Second, they found that backtracking by one base pair is much easier than backtracking by two or more base pairs.

This new model for Pol II bears some resemblance to a model that Andrei Ruckenstein’s group at Rutgers University developed for *E. coli* RNA polymerase without assuming that the translocation step was in equilibrium (Maoiléidigh et al., 2011). Both models produce similar results, and the ‘intermediate pause state’ proposed for *E. coli* plays a similar role in modelling as the one-base-pair backtracking state does for Pol II in yeast.

The work by Bustamante and colleagues—who are based at the University of California Berkeley, the Lawrence Berkeley National Laboratory and the National Cancer Institute Center for Cancer Research—has now brought the importance of kinetic measurements to the forefront. However, more work is needed to work out all the details of the coupling between the chemical and mechanical processes. In particular, this latest Pol II model does not consider the effects of the sequence of bases, even though the elongation velocity is expected to greatly depend on sequence: indeed, if sequence-dependent kinetics is included in a simple model that assumes that the translocation step is in equilibrium, the force-velocity curve predicted by the model agrees with that observed in experiments on *E. coil* RNA polymerase (Bai et al., 2007).

Comparing the various models of transcription will require sequence-dependent measurements that are capable of directly imaging translocation events. Luckily, it appears that advances in single-molecule optical trapping and fluorescence techniques will make such measurements possible in the years ahead.
